# Aneurysmal bone cyst on top of fibro-osseous lesion of the ethmoid sinus with orbital and intracranial extension in a child

**DOI:** 10.1259/bjrcr.20210246

**Published:** 2022-03-07

**Authors:** Ayman Ahmed Elsayed, Rawia Mubarak Hamad Mohamed, John Charles Devine, Jonathan Wasserberg, Mohamed Reda Elbadawey, H. S. S. Abdelsamad, Sameeha Sajid, Zeenah Ryad Mansour

**Affiliations:** 1 Consultant Radiologist, Sheikh Shakhbout Medical City in partnership with Mayo Clinic. Adjunct Professor of Radiology, Faculty of Medicine and Health Sciences, Khalifa University, Abu Dhabi, UAE; 2 Consultant Anatomical pathologist, Sheikh Shakhbout medical city in partnership with Mayo clinic. Associate professor in Pathology, Faculty of Medicine and Health Sciences; Khalifa University, Abu Dhabi, UAE; 3 Consultant Maxillofacial-Head and Neck Surgery, Sheikh Shakhbout Medical City in partnership with Mayo Clinic; Adjunct Professor of Surgery, Faculty of Medicine and Health Sciences, Khalifa University, Abu Dhabi, UAE; 4 Consultant Neurosurgeon. Sheikh Shakhbout Medical City in partnership with the Mayo Clinic, Abu Dhabi, UAE; 5 Consultant ENT surgeon. Sheikh Shakhbout Medical City in partnership with the Mayo Clinic, Abu Dhabi, UAE; 6 Faculty of Medicine and Health Sciences, Khalifa University, Abu Dhabi, UAE

## Abstract

**Objective:**

Aneurysmal bone cysts (ABCs) rarely involve the cranium. We report a case arising in the ethmoid sinus with orbital and intracranial invasion. Imaging suggested an associated fibro-osseous lesion. The lesion was completely resected. Histology confirmed the imaging diagnosis of ABC on top of an ossifying fibroma. A multidisciplinary approach is essential for optimal surgical outcomes.

**Methods:**

We report a case of an 8-year-old boy with a 5 week history of painless, increasing prominence of the left eye. Clinical examination revealed non-pulsatile left proptosis. Visual acuity and ocular movements were normal. CT and MRI scans of the maxillofacial regions showed a large space-occupying lesion involving the left ethmoid air cells with left orbital and left inferior frontal intracranial extension. Multiple fluid levels with blood products were seen. Areas of the bony component of the lesion showed ground-glass density on CT. Imaging was consistent with an ABC with an underlying fibro-osseous lesion; probably fibrous dysplasia which was confirmed after surgical removal of the lesion. A multidisciplinary team of maxillofacial, neurosusugery and ENT surgeons performed the surgery.

**Results:**

ABC arising from an osseous fibroma of the skull is rare. Total resection can be achieved with a multidisciplinary surgical approach. Post-operative histology confirmed by the imaging findings.

## Introduction

Aneurysmal bone cysts (ABCs) were first described in 1942.^1^ The most common location is long bones, and most cases present between early childhood and adolescence. Involvement of the skull, however, is rare.^2^

We report a rare case of a giant ABC associated with a fibro-osseous lesion involving the ethmoid sinus, orbit and anterior cranial fossa. Gross total surgical resection was achieved. Imaging and histopathological features are described.

## History and examination

An 8-year-old boy presented with a 5 week history of painless, increasing prominence of the left eye. Parents noticed the swelling after the child had sustained a minor injury while playing. No visual disturbance was reported. Clinical examination revealed non-pulsatile left proptosis measuring 22 mm by exophthalmometer compared to the normal right eye (18 mm). Visual acuity and ocular movements were normal.

## Imaging

**Figure 1.**
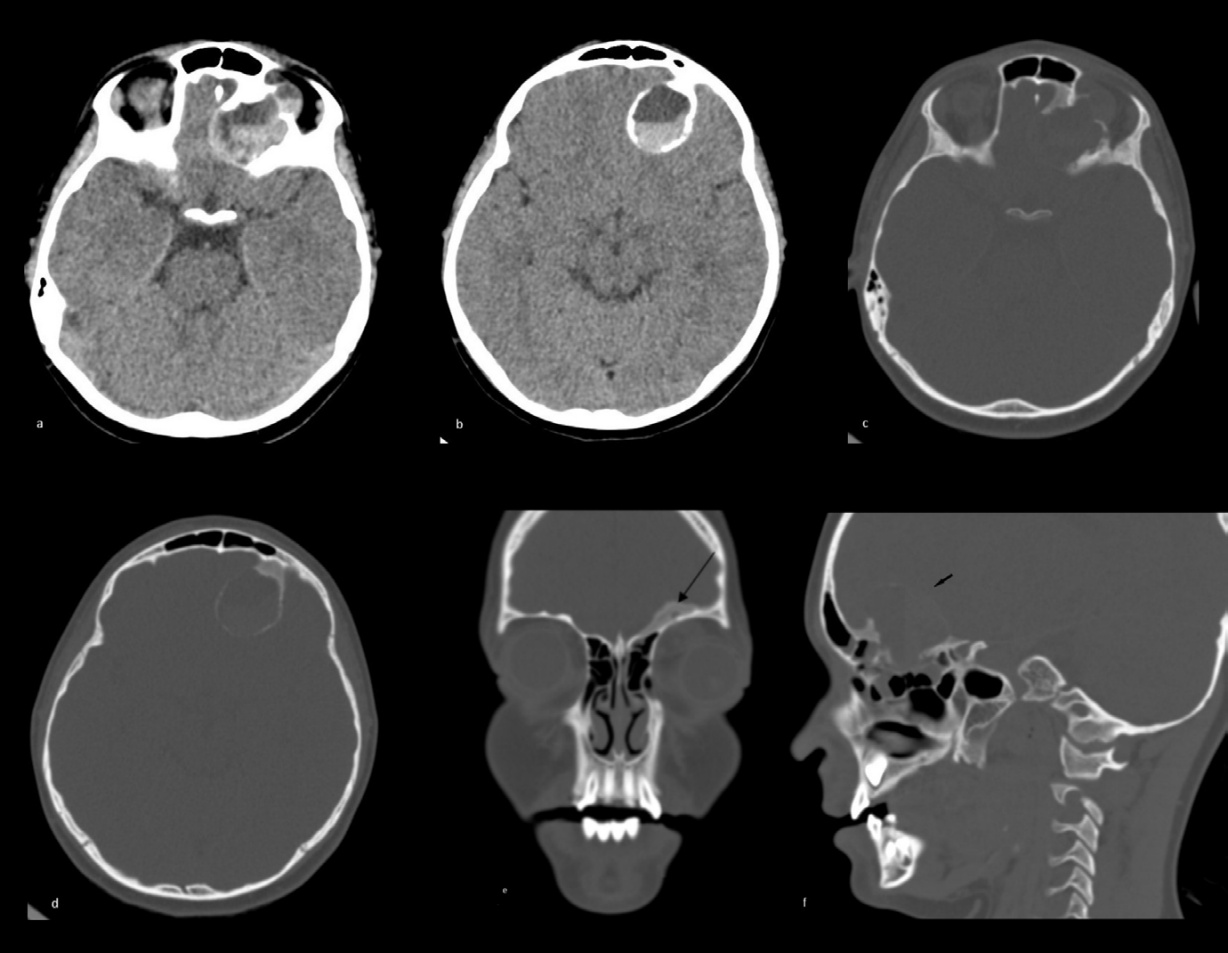
CT head: Soft tissue window (**a, b**) showing a large expanding space occupying lesion involving the left ethmoid air cells with destruction of the left orbital roof and left orbital and left inferior frontal intracranial extension. High-density fluid level indicating blood within the lesion (haematocrit effect). Bone window (**c, d, e, f**) showing calcified rim of the cystic lesion and ground-glass density (thick black arrow) There is a bone defect of the left cribriform plate allowing extension of the lesion intra cranially forming a large extra-axial component with a calcified rim extending under the left frontal lobe (short black arrow).

**Figure 2.**
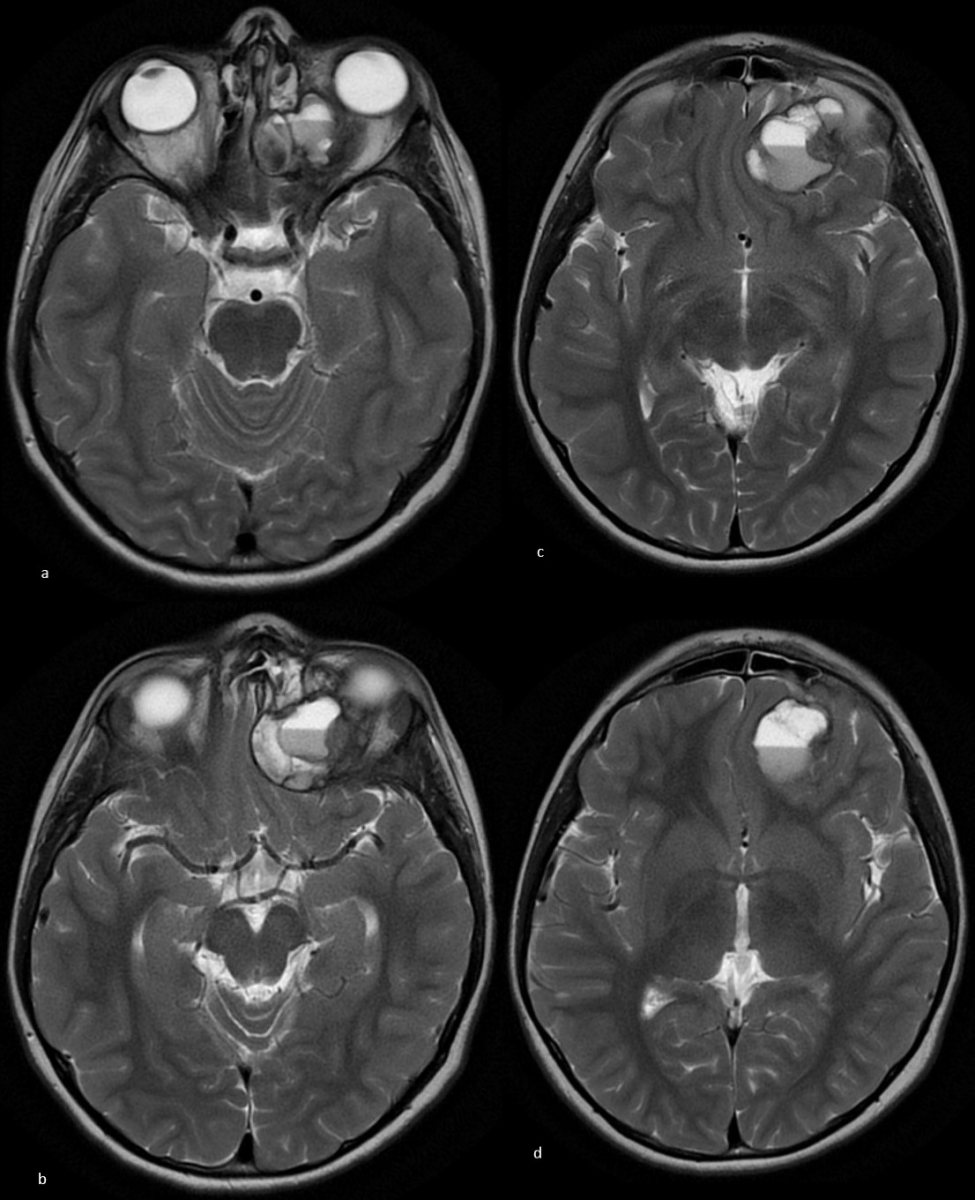
Axial *T*_2_WI (**a**) Mid orbital level showing multiple fluid/fluid levels with dependent dark blood intensity (haematocrit effect). Invasion of the left orbit with superomedial extraconal component. (**b**) High orbital level showing the cystic haemorrhagic lesion invading the left orbit with intracranial extra-axial extension. (**c**)Extra-axial component of the multiloculated septated lesion with mass effect on the left frontal lobe. (**d**) Mass effect on the left frontal lobe with mild midline shift to the right.

**Figure 3.**
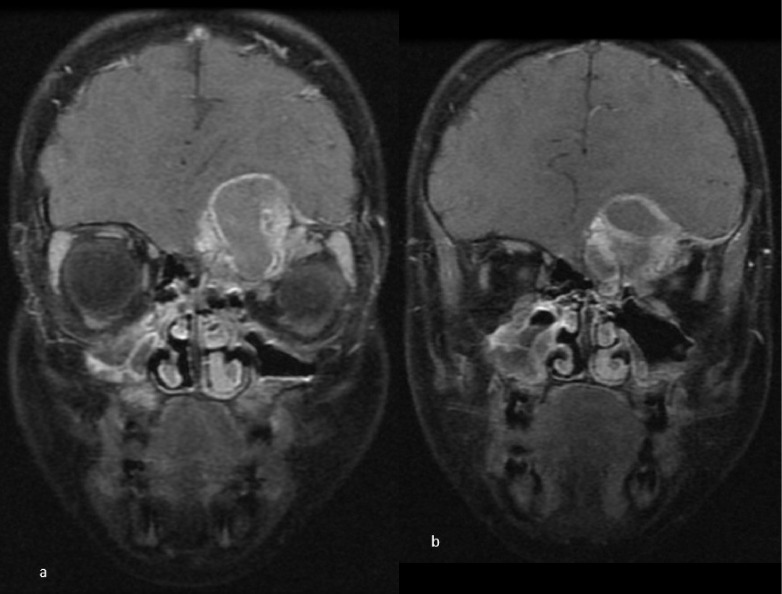
Coronal *T*_1_WI with fat suppression post-Gadolinium showing (**a**) mild enhancement of the thick outline of the lesion and mass effect on the left eye globe inferiorly and the under surface of the left frontal lobe. (**b**) A rim of dural enhancement is noted at the anterior and outer inferior aspect of the left frontal region.

**Figure 4.**
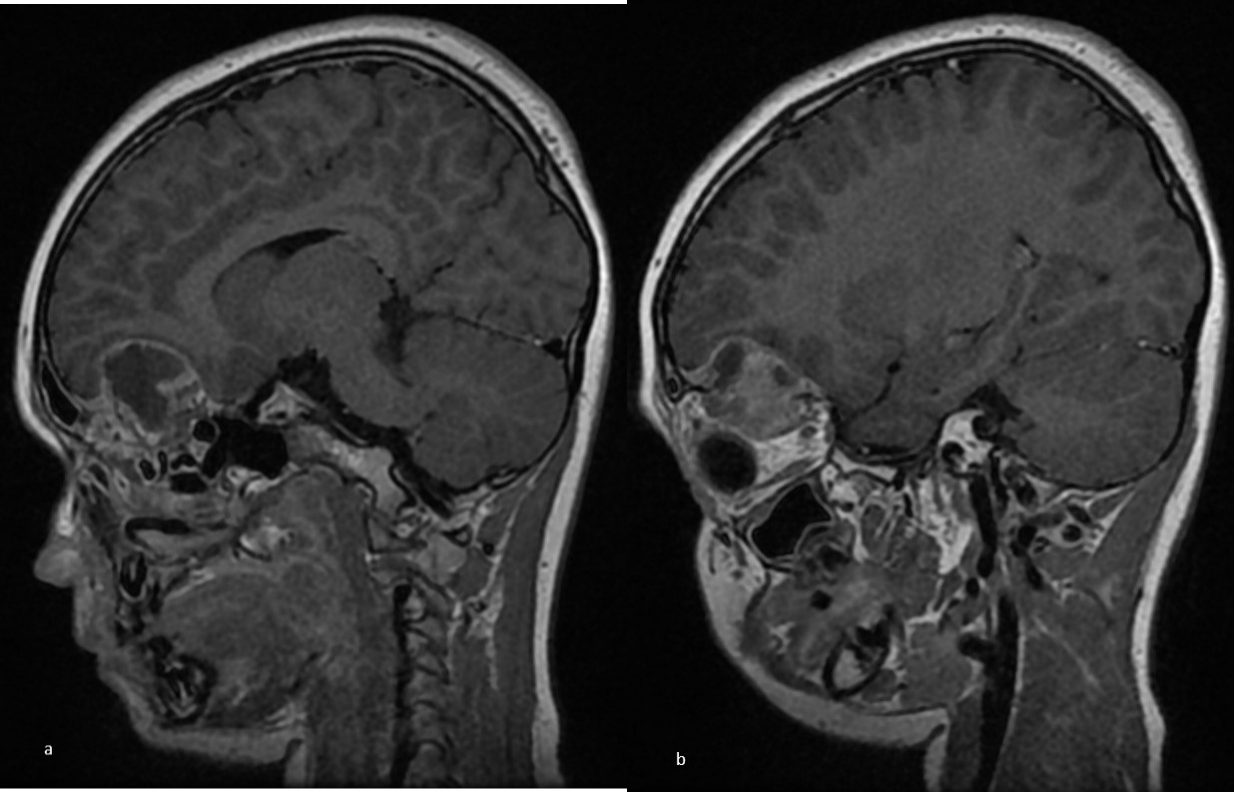
Sagittal *T*_1_WI post-Gadolinium showing (**a**) the intracranial component of the lesion as well as (**b**) the intraorbital component.

CT and MRI (Figures 1–4) demonstrated a large multiloculated septated haemorrhagic left ethmoid space-occupying lesion with left orbital and intracranial extension. The areas of ground-glass density on CT (Figure 1B, D and E) were consistent with an underlying fibro-osseous lesion such as fibrous dysplasia.

## Surgical management

A multidisciplinary team of Maxillofacial, ENT and Neurosurgery performed a total surgical resection of the lesion. Pre-operative nasal endoscopy confirmed origin of the lesion from the left ethmoid sinus and medial wall of the left orbit. A bicoronal flap was raised along with a vascularised pericranial flap and a bifrontal craniotomy was performed to access the lesion. An irregular thick walled fluid filled cystic lesion was seen coexisting with stippled hypervascular irregular bone that was indenting the medial and superior walls of left orbit. The lesion extended into the ethmoids and posteriorly along the orbital roof, encased in thin bone. Using the microscope, plane of cleavage was developed between the lesion wall and the overlying dura mater. To permit further mobilisation, the mass was opened and gelatinous liquid was released decompressing the cyst. Following resection of the lesion, the large anterior fossa skull base defect was repaired with a pedicled nasal septal flap from below and a vascularised pericranial flap from above in conjunction with a durasealant Xact (Integra Life Sciences Corporation, Plainsboro, NJ). The patient made an excellent recovery and was discharged 4 days post-surgery.

## Pathological findings

**Figure 5.**
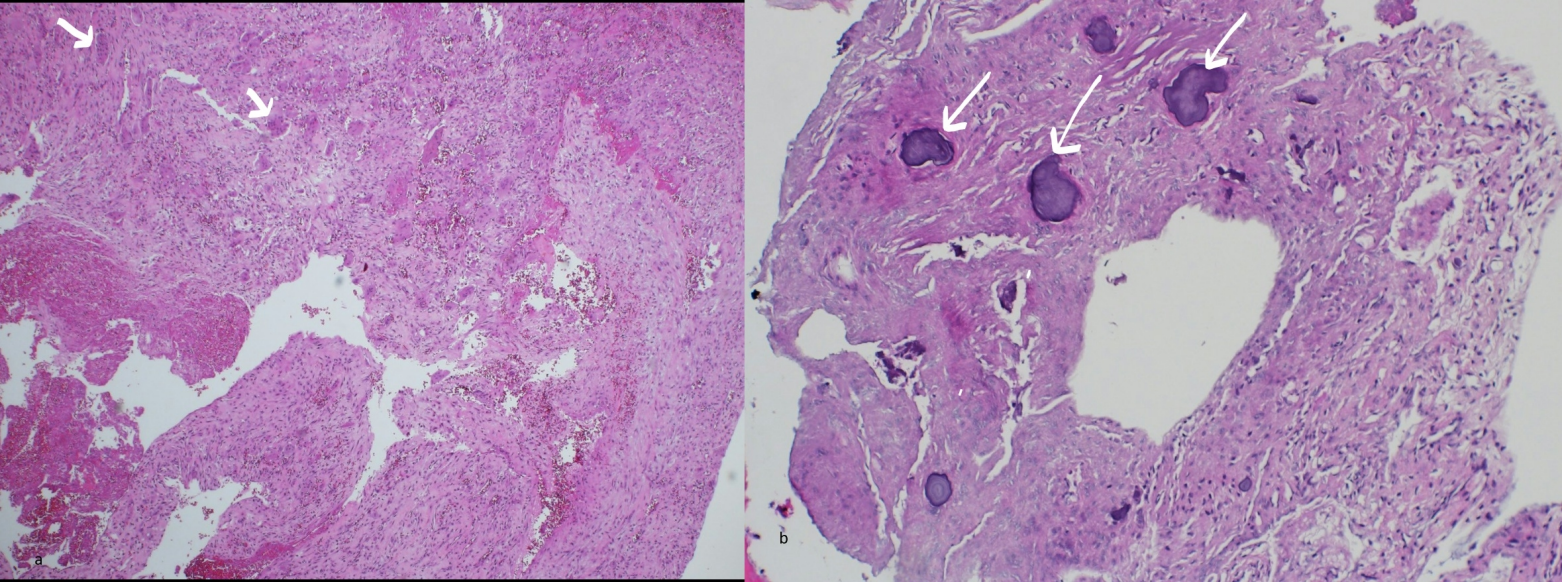
(**a**) Haematoxylin and eosin stain. Low 4x power specimen of bone tissue; cyst wall around the tumour showing spaces filled with RBCs and many multinucleated giant cells (white arrows). (**b**) 10x low power showing cement like material (white arrows) in an area of fibrous stroma. RBC, red blood cell.

Specimens of bone tissue around the tumour, left orbital bone and left frontal bone showed features of ABC with spaces filled with RBCs. The wall of the cyst showed spindle cells with benign cytomorphology, a large number of multinucleated giant cells and cement like material in an area of fibrous stroma. No evidence of malignancy (Figure 5A and B).

## Discussion

ABCs are uncommon lesions, accounting for 2% of all primary bone tumours predominantly involving the metaphysis of long bones.^3^ However, it can affect any bone in the body. Only 3–6% of ABCs affect the skull.^2^ ABCs in the head and neck area are present in only 2% of cases.^4^ The mandible and maxilla are the most frequently involved sites with a mandible predilection of 2:1 over the maxilla^4^ ; with over 90% of cases reported above 30 years of age.^3^ The involvement of the ethmoid bone as in our case is rare.^5^

Clinically, presentation depends on location, size and compressive effects of the mass on surrounding structures.^4^ Neurologic deficits are more likely to happen when the skull base is involved. No neurologic abnormality was found in our patient where painless proptosis was the presenting symptom.

ABCs are classified as primary or secondary, with primary lesions appearing in isolation. Secondary lesions can develop in a number of benign and malignant bone lesions or trauma.^2^ The most common prior lesion is giant cell tumour. Other lesions include fibrous dysplasia, nonossifying fibroma, fibromyxoma, chondroblastoma, osteoblastoma and osteosarcoma.^2^

The pathogenesis of ABCs remains controversial. It may be related to genetic predisposition, post-traumatic or reactive vascular malformation.^6^

Histopathology provides supportive data to confirm the diagnosis of an ABC and distinguishing it from other haemorrhagic bone tumours. Pathology reveals large areas of haemorrhage with fibroblastic septations lacking an endothelium.^6^ This helps distinguish ABCs from hemangiomas. Reports also documented a pathological appearance of woven bone with proliferating fibrous tissues and blood-filled chambers with bony islands.^6^

ABCs are benign locally destructive rapidly growing tumours. In our patient, the ABC is probably secondary to an ossifying fibroma. ABCs secondary to ossifying fibroma remain a relatively uncommon finding in the facial bones.

Few cases in the literature describe a close association of ossifying fibroma with juvenile ABCs as in our case.^3^ Benign fibro-osseous lesions include fibrous dysplasia, ossifying fibroma and cement-osseous dysplasia.

The imaging diagnostic workup for ABCs usually consists of a CT scan, followed by an MRI. CT usually shows a heterogeneous mass with solid and cystic regions.^7^ Reports state that 87% appear as radiolucent and only 2% are radiopaque with the rest having mixed opacity.^7,8^ Other findings also include ground-glass density, osseous expansion, narrowing of the foramina and contrast enhancement.^8^

Fluid levels are present on CT in 35% of cases, with increased attenuation of the dependent level.^9^ The fluid level is suggestive of a mixture of proteinaceous and blood products.^7^ The mass also presents with associated bony remodelling, as well as areas of non-aggressive bony resorption.^8^

MRI shows fluid levels better than CT.^9^ However, fluid levels may also be seen in simple bone cysts, soft tissue cavernous hemangiomas and cystic hygromas.^10^ Other MRI findings include multiple internal septations and lobulations with varying blood degradation intensities.^9^

However, imaging findings alone are not enough to distinguish ABCs from other pathologies. Differential diagnoses include fibrous dysplasia, haemorrhagic cyst, giant cell reparative granuloma, metastasis and plasmacytoma.^8^

Fibrous dysplasia like ABC is also an expansile mass frequently involving the ethmoid sinuses, but it shows ground-glass appearance on CT.^8^ Telangiectatic osteosarcoma is much more aggressive in nature and appears in older age groups.^8^ Plasmacytoma shows a diffuse homogeneous enhancement after i.v. contrast. Metastatic lesions characteristically show ring enhancement with gadolinium.^8^

Treatment of choice is gross total resection^10^ which is generally curative. This may be more difficult with lesions involving the skull base.

## Conclusion

ABCs of the skull are rare. Involvement of the ethmoid bone in the setting of fibro-osseous lesion as in our patient has been reported in only a handful of previous case reports. Lesions can often be identified based on characteristic imaging findings of multi loculated septated haemorrhagic mass with fluid levels. Areas of ground-glass density on CT suggest the underlying fibro-osseous lesion. CT and MRI help pre-operative anatomical location and extent of intracranial and orbital involvement. Histological examination is essential to make an accurate diagnosis.

## Learning points

- Patients presenting with proptosis should be investigated by cross-sectional imaging.
- Imaging findings of multiloculated septated haemorrhagic mass with fluid levels are highly suggestive of ABC, but other pathologies cannot be ruled out.
- Areas of ground-glass density on CT suggest the underlying fibro-osseous lesion.
- CT and MRI provide pre-operative anatomic details of the ABC as well as invasion of surrounding structures.
- Histological examination is essential to make an accurate diagnosis.
